# Conformational changes in tubulin upon binding cryptophycin-52 reveal its mechanism of action

**DOI:** 10.1016/j.jbc.2021.101138

**Published:** 2021-08-28

**Authors:** Elif Eren, Norman R. Watts, Dan L. Sackett, Paul T. Wingfield

**Affiliations:** 1 Protein Expression Laboratory, NIAMS, National Institutes of Health, Bethesda, Maryland, USA; 2 Division of Basic and Translational Biophysics, NICHD, National Institutes of Health, Bethesda, Maryland, USA

**Keywords:** LY355703, cryptophycin-1, cryptophycin-52, cyclodepsipeptide, microtubule, tubulin, maytansine site, anticancer drug, cryo-EM, conformational change, Cp, cryptophycin, Cp-1, cryptophycin-1, Cp-52, cryptophycin-52, MD, molecular dynamics, MICEF, Multi-Institute Cryo-EM Facility, PDB, Protein Data Bank

## Abstract

Cryptophycin-52 (Cp-52) is potentially the most potent anticancer drug known, with IC_50_ values in the low picomolar range, but its binding site on tubulin and mechanism of action are unknown. Here, we have determined the binding site of Cp-52, and its parent compound, cryptophycin-1, on HeLa tubulin, to a resolution of 3.3 Å and 3.4 Å, respectively, by cryo-EM and characterized this binding further by molecular dynamics simulations. The binding site was determined to be located at the tubulin interdimer interface and partially overlap that of maytansine, another cytotoxic tubulin inhibitor. Binding induces curvature both within and between tubulin dimers that is incompatible with the microtubule lattice. Conformational changes occur in both α-tubulin and β-tubulin, particularly in helices H8 and H10, with distinct differences between α and β monomers and between Cp-52-bound and cryptophycin-1-bound tubulin. From these results, we have determined: (i) the mechanism of action of inhibition of both microtubule polymerization and depolymerization, (ii) how the affinity of Cp-52 for tubulin may be enhanced, and (iii) where linkers for targeted delivery can be optimally attached to this molecule.

Agents such as paclitaxel (Taxol) and the vinca alkaloids such as vinblastine (Velban) that perturb the finely tuned dynamic instability of microtubules are among the most successful of anticancer drugs. They fall into two broad categories, microtubule-stabilizing agents and microtubule-destabilizing agents, which target six sites on the tubulin dimer. For a review, see Steinmetz and Prota (1). More recently, a seventh pharmacologic site has also been defined by gatorbulin-1 (2). Maytansine, a microtubule-destabilizing agent, occupies and defines a site on β-tubulin at the interdimer interface that is also the target of four other agents (rhizoxin, spongistatin, disorazole Z, and phase 1 drug PM060184) (3). To date, only the contacts on β-tubulin have been defined for this site, in part the result of employing stathmin-4 as a crystallization chaperone.

The cryptophycins (Cps) are a class of macrocyclic depsipeptide natural products derived from species of marine cyanobacteria in the genus *Nostoc*. Cps destabilize microtubules, thereby preventing correct mitotic spindle formation and inhibiting cell proliferation (4, 5). They are among the most potent antiproliferatives known, some with IC_50_ values in the single-digit picomolar range, making them 100 to 1000 times more potent than paclitaxel and vinblastine (4). They are also effective against multidrug-resistant cancer cell lines and solid tumors (4). Accordingly, a large number of derivatives have been investigated. Cryptophycin-52 (Cp-52), a biologically more stable analog of the parent compound cryptophycin-1 (Cp-1), has progressed to phase 2 clinical trials for use against both advanced non–small-cell lung cancer (6) and platinum-resistant ovarian cancer (7), and although disease stabilization was observed, the studies were discontinued because of neurotoxicity. Despite this, intense research has continued to identify safer and even more potent synthetic analogs (4, 5) and on antibody- and peptide-targeted delivery (8, 9). However, progress has been hampered by the fact that the binding site is not known, preventing the rational introduction of chemical modifications and the identification of optimal attachment points of linkers for targeted delivery.

At high concentrations, Cp-1 and Cp-52 destabilize microtubules and cause the formation of curved tubulin complexes and rings (10). A majority of these rings have 8-fold symmetry with two points of curvature (11). We have observed that such rings readily form square arrays and crystals but also that these did not diffract well. Therefore, we have determined the structures of Cp-1–HeLa tubulin and Cp-52–HeLa tubulin rings, to a resolution of 3.4 and 3.3 Å, respectively, by cryo-EM and further characterized the binding interactions by molecular dynamics (MD). In our Cp-1–HeLa tubulin and Cp-52–HeLa tubulin samples, we have also observed the formation of rings with 9-fold symmetry (C9) ranging from 24% to 40%. We also solved the structure of Cp-1 C9 rings to a resolution of 3.8 Å. Cp-1 and Cp-52 bind primarily to a site on β-tubulin that overlaps with, but is distinct from, that of maytansine as well as those of rhizoxin and PM060184. The site is also different from ones that have previously been proposed for Cp-52 based on modeling (10, 12). Cp-1 and Cp-52 also interact with α-tubulin, explaining previously observed structure–activity relationships and suggesting new modifications including linkers for targeted delivery. Finally, the location and consequences of binding suggest a mechanism for how Cp-52 reduces microtubule dynamic instability resulting in the antiproliferative effect.

## Results

### Binding sites Cp-1 and Cp-52

#### Overall orientation of Cp-52 on tubulin

The interactions of Cp-1 and Cp-52 with tubulin are very similar at the current resolution. For clarity, the interactions of Cp-52 are described here, whereas differences between Cp-1 and Cp-52 are indicated as necessary. Data collection, processing, and refinement statistics are given in Table 1. Representative images and class averages, Fourier-shell correlation curves and angular orientation distribution plots, local resolution maps, and examples of the electron density maps at the Cp-1 and Cp-52 binding sites are shown in Figures S1–S4, respectively.

**Table 1:** Data collection, processing, and refinement statistics

Deposition Id	EMD-23569	EMD-23615	EMD-23627
	PDB: 7LXB	PDB: 7M18	PDB: 7M20
Data collection			
Magnification	130,000	130,000	130,000
Voltage (kV)	300	300	300
Total dose (e^−^/Å)	66	66	66
Frame rate	4 frames/s	4 frames/s	4 frames/s
Defocus range (μm)	−0.6 to −2.6	−0.6 to −2.6	−0.6 to −2.6
Pixel size	1.06	1.06	1.06
Data processing			
Symmetry	C8	C8	C9
Initial particle number	40,746	35,113	35,113
Final particle number	31,098	18,899	14,468
Resolution (Å)	3.3	3.4	3.8
Fourier shell correlation threshold	0.143	0.143	0.143
Refinement			
Map sharpening *b*-factor (Å^2^)	−90	−90	−150
Model composition			
Nonhydrogen atoms	54,280	54,064	60,705
Protein residues	6928	6904	7785
Ligands	24	24	27
B-factors (Å^2^)			
Protein	125.8	118.6	161.6
Ligand	93.4	84.6	129.1
Nucleotide (GTP/GDP)	119.0/113.9	99.0/95.2	145.0/135.0
RMSD			
Bond length (Å)	0	0	0
Bond angles (°)	0	0	0
Validation			
Model-to-map fit CC protein	0.75	0.73	0.76
Model-to-map fit CC ligand	0.77	0.74	0.69
Model-to-map fit CC nucleotide (GTP/GDP)	0.81/0.76	0.84/0.72	0.77/0.72
Molprobity score	2.45	2.72	2.75
Clashscore	22	21	30
Side-chain outliers (%)	0.9	0.8	3.1
Ramachandran plot			
Favored (%)	87.2	82.9	83.1
Allowed (%)	12.2	16.8	16.6
Disallowed (%)	0.6	0.3	0.3

**Figure 1:**
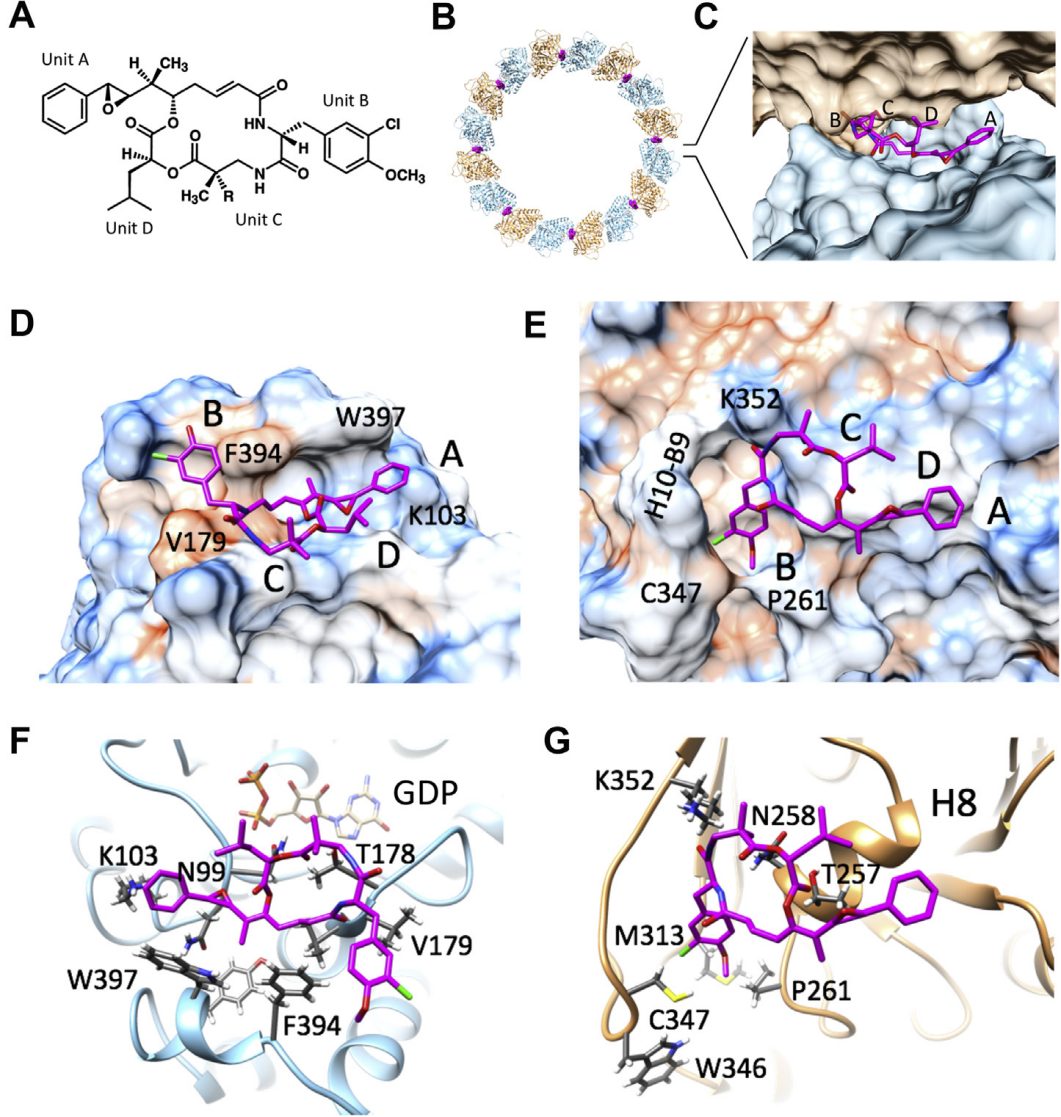
**Binding site of Cp-52 on HeLa tubulin.***A*, structure of Cp with the units A, B, C, and D indicated. In Cp-1, there is a single methyl instead of a *gem*-dimethyl group in unit C (*i.e.*, R = H in Cp-1, CH3 in Cp-52). *B*, tubulin ring with 8-fold symmetry induced by Cp-52; α-tubulin (*brown*), β-tubulin (*blue*), and Cp-52 (*magenta*). *C*, view of Cp-52 (stick, *magenta* and heteroatom) bound at the maytansine site between β-tubulin (*blue*, surface) and α-tubulin (*brown*, surface) as viewed from outside the ring. *D*, Cp-52 (stick, *magenta* and heteroatom) interaction with β-tubulin (hydrophobicity surface). *E*, Cp-52 (stick, *magenta* and heteroatom) interaction with α-tubulin (hydrophobicity surface). *F*, Cp-52 (stick, *magenta* and heteroatom) interaction with β-tubulin (ribbon, *blue*), as viewed toward the minus-end. GDP is indicated. *G*, Cp-52 (stick, *magenta* and heteroatom) interaction with α-tubulin (ribbon, *brown*), as viewed toward the plus-end. Helix H8 is indicated. Cp-52, cryptophycin-52.

**Figure 2:**
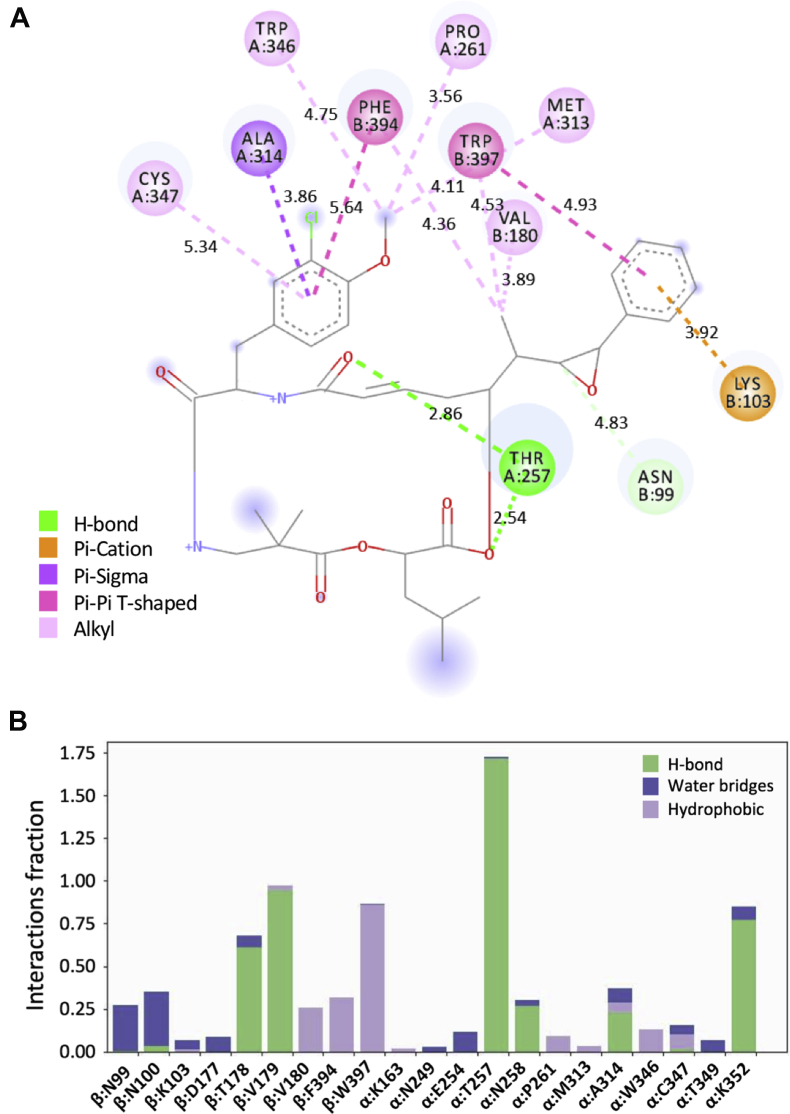
**Interactions of Cp-52 with β-tubulin and α-tubulin.***A*, 2D schematic of interactions with different residues. *B*, interaction fractions with different residues, as determined by molecular dynamics. Cp-52, cryptophycin-52.

Cp-52 is composed of four structural units, A to D (Fig. 1*A*). Cp-52 induces tubulin to form rings consisting of eight tubulin heterodimers (Fig. 1*B*). The cryo-EM reconstruction shows that Cp-52 is bound at the interdimer site, immediately adjacent to the contact region between β-tubulin and α-tubulin, and oriented with unit A toward the right, unit B toward the left, and units C and D toward the viewer, when the ring is viewed from the outside as shown (or when a microtubule protofilament is viewed from the luminal side with the plus-end up) (Figs. 1*C* and S5). The phenyl group of unit A and the isobutyl group of unit D are exposed, whereas the aromatic ring of unit B is in a deep pocket between β-tubulin and α-tubulin. This difference in exposure is reflected in the MD analysis, where units A and D have large positive root-mean-square fluctuation peaks, whereas unit B has a negative peak (Figs. 2 and S5 and Movie S1).

#### Detailed contacts of Cp-52 with β-tubulin

Cp-52 interacts primarily with β-tubulin. It is positioned in a partially hydrophobic groove on the tip of the β-subunit such that the aromatic ring in unit A forms a strong pi–pi T-interaction with β:W397 and a pi–cation interaction with β:K103 (Figs. 1, *D* and *F* and 2). The pi–cation interaction with β:K103, and the dependence on the benzyl quadrupole for this interaction, may explain the activity sensitivity of this group to even subtle modification, in some instances up to several orders of magnitude (5). The epoxide in unit A is oriented toward β:N99, and the adjacent methyl group projects into a pocket formed by β:V180, β:F394, β:W397, and β:Y398, where it forms alkyl and pi–alkyl interactions (Figs. 1, *D* and *F* and 2). The depth of this pocket suggests that the methyl group could potentially be replaced with a slightly larger hydrophobic one. The aromatic ring on unit B forms a pi–pi T-interaction with β:F394. The carbonyl groups on the macrocyclic core form hydrogen bonds with β:N100, β:T178, and especially β:V179 (Figs. 1, *D* and *F* and 2).

#### Detailed contacts of Cp-52 with α-tubulin

Cp-52 unit B fits into a pocket on α-tubulin formed by the C-terminal end of α:H8, the N-terminal end of α:B8, and the strand between α:H10 and α:B9 (Fig. 1, *E* and *G*). The aromatic ring on unit B makes contacts with α:A314 and α:C347, whereas the methoxy group engages in alkyl interactions with α:P261, α:M313, and α:W346 (Fig. 1, *E* and *G*). The carbonyl groups on the macrocyclic core form hydrogen bonds especially with α:T257, but as shown by MD, also α:N258, α:A314, and α:K352 (Figs. 1, *E* and *G* and 2). The contact area of Cp-52 with β-tubulin is 380.9 Å^2^ (339.2 Å^2^ for Cp-1), similar to that of maytansine (313.2 Å^2^) and rhizoxin (333.6 Å^2^) but less than that of PM060184 (489.3 Å^2^). The contact areas of Cp-1 and Cp-52 with α-tubulin are 339.0 and 373.0 Å^2^, respectively. The contacts of maytansine, rhizoxin, and PM060184 with α-tubulin are not known. The calculated dissociation constants for Cp-1 and Cp-52 are p*K*_d_ = 7.24 and p*K*_d_ = 7.09, respectively.

### Binding of Cp-1 and Cp-52 induces conformational changes in α-tubulin and β-tubulin

#### Geometry of rings

Cp-52 induces tubulin to form rings consisting of eight tubulin heterodimers (Fig. 1*B*). As observed previously, such rings have two points of curvature indicating both intradimer and interdimer bending (11). Relative to the straight conformation in microtubules, the intradimer and interdimer angles are, on average, 12.1° and 35.6°, respectively (Table S1). The interface areas are 62% and 32%, respectively, relative to those in microtubules. The interface residues at the intradimer and interdimer sites in the rings are a subset of those in microtubules; no new residues are involved in forming these interfaces, although the degree of burial of the side chains is different (not shown). Key residues (buried surface area >40%) at the interfaces are the same for both Cp-1 and Cp-52. The interaction energies at the intradimer and interdimer sites in Cp-52 rings are −15.3 and −7.8 kcal/mol, respectively. For comparison, the interaction energies at the corresponding sites of microtubules are −26.87 ± 7.05, n = 6 and −20.06 ± 4.46, n = 6 kcal/mol, respectively (Table S1). The significance of the small differences in interaction energies between Cp-1-bound and Cp-52-bound tubulin is uncertain at the current resolution.

#### Conformational changes in tubulin subunits

**Figure 3:**
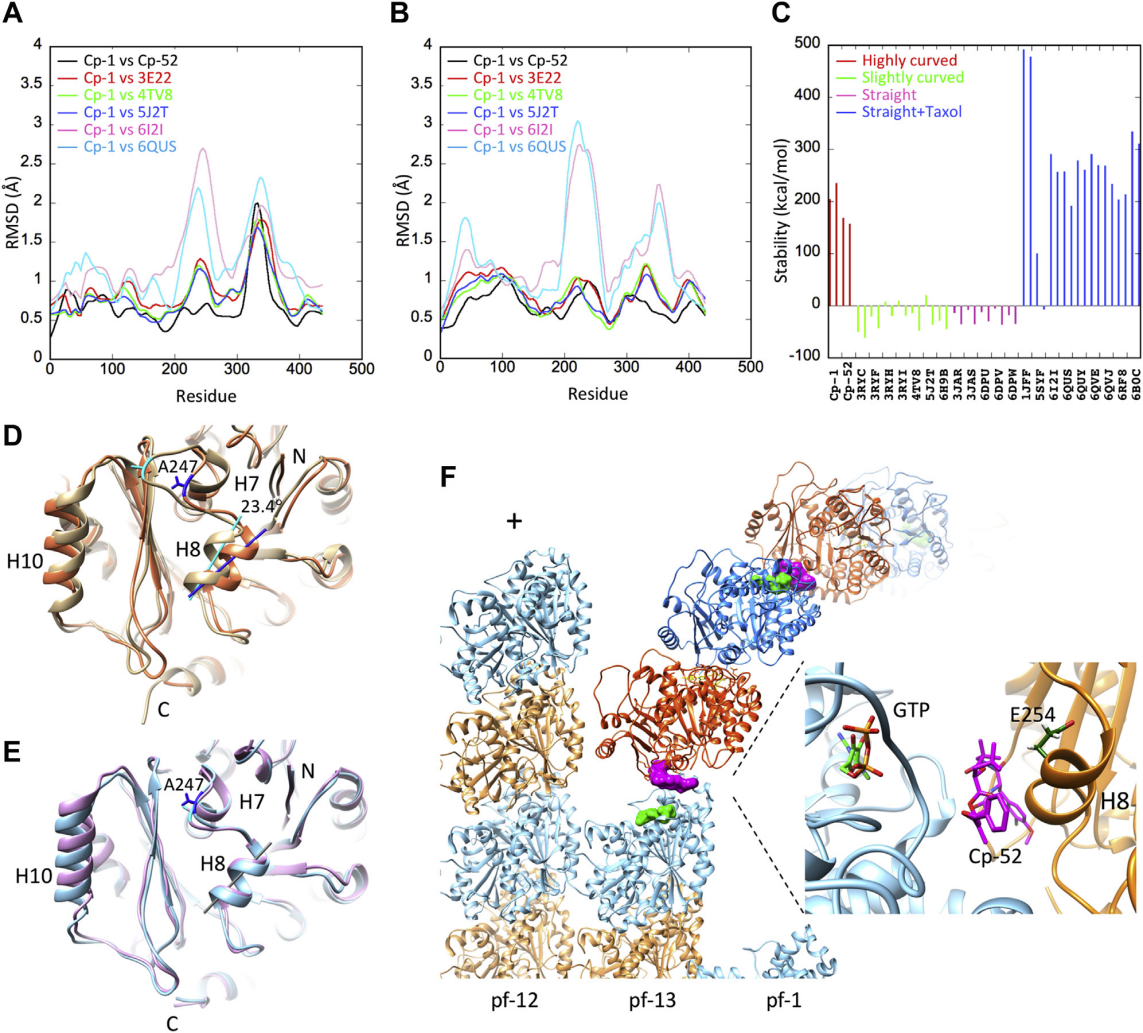
**Conformational changes in α-tubulin and β-tubulin upon Cp-52 binding and ring formation.***A*, per-residue Cα-RMSD between α-tubulin subunits in complexes with different degrees of curvature: Cp-1-bound and Cp-52-bound (*black*) tubulins, Cp-1-bound and colchicine–soblidotin–stathmin-bound (*red*) tubulins, Cp-1-bound and maytansine–stathmin-bound (*green*) tubulins, Cp-1-bound and vinblastine–stathmin-bound (*blue*) tubulins, and Cp-1-bound and Taxol-bound (pink and cyan) tubulins. Cp-1-bound and Cp-52-bound tubulin is highly curved, colchicine–soblidotin–stathmin-bound tubulin, maytansine–stathmin-bound tubulin, and vinblastine–stathmin-bound tubulin is slightly curved, and Taxol-bound tubulin is not curved. Plot curve smoothing is 10%. *B*, per-residue Cα-RMSD between β-tubulin subunits in complexes with different degrees of curvature. Complexes are otherwise as in (*A*). *C*, stabilities of subunits in highly curved Cp-1-bound and Cp-52-bound rings (*red*), slightly curved complexes (*green*), microtubules (*magenta*), and Taxol-bound microtubules (*blue*). The stabilities of α-tubulin and β-tubulin are shown for each structure, *left and right columns*, respectively. Stability values are calculated with FoldX. *D*, conformational differences between HeLa α-tubulin in a microtubule (Protein Data Bank ID: 6I2I, *tan*) and HeLa α-tubulin in a Cp-52-bound ring (*brown*). α:H8 (residues 252–259) in the ring is rotated 23.4° clockwise relative to that in the microtubule, as viewed toward the protofilament plus-end. The T7 loop at α:A247 is also retracted 7 Å. *E*, conformational differences between HeLa α-tubulin in Cp-1-bound (*lilac*) and Cp-52-bound (*blue*) rings. The structures are essentially identical, including at α:H8 (the two helix axes are coincident) and the T7 loop but differ at α:H10 where that of Cp-52 is shifted 2.4 Å toward the exterior side of the subunit. *F*, model of Cp-52 mechanism. Plus-end of a microtubule with a 13-3 B-lattice viewed at the seam from the lumen. Microtubule (α-tubulin, *brown*; β-tubulin, *blue*), incoming tubulin dimer (α-tubulin, *orange*; β-tubulin, *dodger blue*), Cp-52 (*magenta*), and GTP (*green*). Note, the nucleotide in our structures has GDP at the exchangeable site (see Experimental procedures section), but on a microtubule in a cell, this would be GTP. Only select ligands are shown, for clarity. Cp-52, cryptophycin-52.

Tubulin dimers undergo conformational changes upon binding Cp-1 and Cp-52 and ring formation. RMSD plots reveal larger conformational differences between the dimers in highly curved Cp-52–tubulin rings and those in microtubules and smaller differences relative to slightly curved tubulin–stahmin-4 complexes. These differences occur in both α-tubulin and β-tubulin (Fig. 3, *A* and *B*). It should be noted that the differences in the RMSD plots appear to be less because of smoothing. The greatest differences occur between the regions including H7 (residues 224–241) and especially H8 (residues 252–259), and H10 (residues 325–335). The H8 helices, in both the α subunit and β subunit, in both Cp-1-bound and Cp-52-bound tubulin, undergo a rotation relative to their counterparts in microtubules. Relative to three microtubule reference structures, α:H8 in Cp-52-bound tubulin is rotated an average of 23.4° clockwise, as viewed toward the protofilament plus-end (Fig. 3*D*). This reorientation accommodates the binding of Cp-52 between β-tubulin and α-tubulin. There are also conformational differences between Cp-1-bound and Cp-52-bound tubulin in that α:H10 of the latter is shifted out of register, relative to the former, by 2.4 Å (*i.e.*, half a turn) toward the exterior of the protofilament (Fig. 3*E*). This shift does not occur in β:H10, likely because it is distal to Cp-52, which is bound near α:H10.

#### Subunit stabilities

The conformational differences discussed previously are reflected in the stabilities of the subunits (Fig. 3*C*). The average calculated stability of the α subunits and β subunits in Cp-1-bound and Cp-52-bound tubulin rings is +191.4 ± 35.5, n = 4 kcal/mol. By comparison, the average stability of the two subunits in 12 different reference structures (five microtubules and seven slightly curved ones) is −23.5 ± 19.9, n = 24 kcal/mol. However, in nine additional microtubules stabilized with Taxol, the average stability of the subunits is +262.5 ± 113.5, n = 18 kcal/mol. Across all 23 dimeric structures, the β-subunit is on average 22% more stable than the α subunit. It appears that binding of Cp-1 and Cp-52 and forming rings results in conformational strain almost as great as that associated with binding Taxol.

A previous MD analysis has shown that tubulin dimers exist in a continuum of intermediate bent states (13). Intradimer bending was found to be associated with not only intersubunit but also intrasubunit rotations (*i.e.*, a twist). Free energy profiles showed a global minimum at an intradimer bending angle of ca. 6° and maxima at ca. 0° (intact microtubule lattice) and ca. 12°, the latter value being similar to the 12.1° intradimer angle observed here. Image classification indicates that the majority (ca. 2/3 and 3/4 of the Cp-1-bound and Cp-52-bound particles, respectively) have 8-fold symmetry, most of the remainder have 9-fold symmetry (Fig. S6), and a very small fraction appear to have lower symmetries (not shown). Taken together, these observations suggest that a 12.1° intradimer angle is the maximum possible, that any further bending must occur at the interdimer interface, and that 35.6° is close to the maximum possible at this site. It may be that the conformational energy cost associated with even higher curvature is prohibitive, that the energy of association between the subunits becomes too little, or that an even greater interdimer bending angle no longer allows formation of the binding site.

## Discussion

Cp-52 is potentially the most potent antiproliferative known. Exceedingly low concentrations of Cp-52 inhibit cell proliferation at mitosis (IC_50_ = 11 pM) without substantially altering either microtubule length or mass (14). It has also been shown that Cp-52 binds very tightly to tubulin, and that the affinity of the Cp-52–tubulin complex for microtubules is very high (*K*_d_ = 47 nM). As low as 20 nM Cp-52, and as few as five to six Cp-52 molecules per microtubule end, decrease dynamicity (the rate of total tubulin dimer exchange at a microtubule end) by 50% (14). As microtubules grow and depolymerize primarily at the plus-end, ca. five molecules are sufficient to modulate a microtubule's dynamics.

### Cp-52 mechanism of action

We have found that Cp-52 binding induces tubulin to form rings with an interdimer angle of 35.6° and an intradimer angle of 12.1°. The large interdimer angle is necessary to accommodate Cp-52, whereas the smaller intradimer angle may only be a consequence of ring closure. When such rings are aligned to microtubule structures (*e.g.*, Protein Data Bank [PDB] ID: 5SYF), at their respective β-subunits, they do so at an angle of ca. 73° (71° for Cp-1 rings) relative to the surface, counterclockwise as viewed toward the plus-end. Alignment of a ring to protofilament-13 (*i.e.*, adjacent to the seam) at the plus-end shows that the large angle formed between a terminal dimer and an incoming dimer by the binding of Cp-52 is incompatible with the microtubule lattice (Fig. 3*F*). It not only orients the incoming dimer out of the microtubule wall but also away from the adjacent protofilament. This would prevent any further addition of dimers, and if this were to also occur at adjacent protofilaments, microtubule growth would halt. At the same time, binding of Cp-52 blocks access of the catalytic E254 in α:H8 (15) of the incoming dimer to GTP on the terminal β-subunit (Fig. 3*F*, inset). This would prevent hydrolysis, preserve the GTP cap, and stabilize the microtubule against depolymerization. Both growth and depolymerization depend on the dissociation of Cp-52–tubulin complexes from the plus-end. In this way, only a very few Cp-52 molecules could decrease both microtubule growth and shortening, as previously reported (14).

### Potential modifications of Cp-52

The structure–activity relationship studies of Cp have been recently reviewed (16). Most changes have been found to be deleterious, and it has also proven difficult to find attachment points for linkers. These observations can be understood now that the binding site is known. In unit A, *para*-substituted phenyls are more potent than *ortho-*substituted or *meta*-substituted analogs, likely because the *para* position is relatively exposed, whereas the latter two and particularly the *ortho* positions would have greater steric constraints. This also explains why the *para* position can serve as a linkage point. The preference for the β configuration over the α configuration of the benzylic epoxide may rest in the greater complementarity of the former to the surface of the β-tubulin subunit. In unit B, the sensitivity to slight modifications, even a 5-fluorine substitution on the benzene ring, reflects the tight pocket that this group is located in. In unit C, bulky substituents such as benzyl or isopropyl groups at the C6 position strongly reduce potency, likely because of steric clash in the constriction between the β-tubulin and α-tubulin subunits at this point. In unit D, only slight changes in potency occur following substitution, in agreement with the observation here that this group is quite exposed.

There appear to be two points where the structure could be altered. In unit A, the methyl group adjacent to the epoxide could be extended slightly to better occupy the pocket formed by β:V180, β:F394, β:W397, and β:Y398 to increase the affinity. In unit D, the isobutyl group could serve as a linker attachment point. Linkers would need to be extended (>30 Å), of low bulk, with consideration given to the proximity of α:E251 and α:E254 to the attachment point. Linkers attached at the unit A aromatic ring have the potential to deleteriously affect both the electronic and steric characteristics of this group. Unlike the unit D isobutyl group, which is readily accessible from the luminal side of the protofilament, the unit A phenyl group is poorly accessible, particularly from the direction of the adjacent protofilament. Linkers with greater residual bulk after cleavage are likely to be similarly affected. Therefore, linkages through the unit D isobutyl group have the potential to be superior to those through the unit A phenyl group. Linkers attached by opening the unit A epoxide ring are also likely sterically hindered. In summary, we have determined the binding site of Cp on tubulin. This suggests both its mechanism of action and ways to enhance the efficacy as well as the specificity of the drug.

## Experimental procedures

### Preparation of HeLa tubulin and formation of Cp-1–tubulin and Cp-52–tublin ring complexes

HeLa tubulin was prepared according to the method described (17). HeLa cells were obtained from Accurate Chemical and Scientific Corp. Cells were lysed by sonication in an equal volume of PME buffer (0.1 M 1,4-piperazinediethanesulfonic acid, 1 mM MgCl_2_, 1 mM EGTA, and pH 6.9). A 100,000*g* supernatant was prepared and absorbed on a DEAE Sepharose Fast Flow column, washed with PME buffer, followed by 0.2 M sodium glutamate in PME buffer, and eluted with 1 M sodium glutamate in PM buffer. Fractions of 1 g/l or greater were combined, and polymerization was induced by addition of 1 mM GTP and 8% v/v dimethylsulfoxide and warming to 37 °C for 30 min. Microtubules were collected by centrifugation at 100,000*g* and depolymerized in PM buffer on ice, followed by a short 80,000*g* centrifugation. The supernatant was drop frozen and stored in liquid nitrogen. This tubulin has GDP at the exchangeable site.

Cp rings were formed by thawing the tubulin, removing minimal aggregates by a 5-min centrifugation at 100,000*g*, diluting it to the desired concentration (*e.g.*, 5 μM), and addition of Cp stock (in dimethylsulfoxide) to 10 μM. Samples were then incubated at 37 °C for 10 min followed by 15 to 20 min at room temperature.

### Structure determination

#### Cryo-EM sample preparation and data acquisition

Three microliters of the samples at 0.5 mg/ml were applied to Quantifoil R1.2/1.3300 mesh Cu grids coated with 2-nm ultrathin carbon (Electron Microscopy Sciences, Protochips, Inc), which were previously plasma cleaned for 12 s with an argon–oxygen mixture (25% oxygen) in a model 1020 plasma cleaner (Fischione). Using a Leica EM GP plunger (Leica Microsystems), excess liquid was blotted 4 to 6 s after a 60-s wait time (95% humidity, 4 °C), and grids were flash frozen in liquid ethane.

Data collection was performed with a Titan Krios G3 cryo-electron microscope (Thermo Fisher Scientific) operated at 300 kV at the Multi-Institute Cryo-EM Facility (MICEF; National Institutes of Health). Micrographs were recorded as dose-fractionated movies with a Gatan K2 Summit direct electron detector operated in counting mode at a nominal magnification of 30,000× (calibrated pixel size of 1.06 Å). The total dose for each exposure was 66 e-/Å^2^, where the total exposure time was 10 s fractionated into 40 frames with 0.25-s exposure time for each frame. The nominal defocus range used was −0.6 to −2.6 μm. For Cp-1–tubulin and Cp-52–tubulin samples, a total of 2403 and 2592 movies were acquired, respectively, using SerialEM (18).

#### Image processing

The movies were aligned using Bsoft (19). Contrast transfer function estimations were carried out using CTFFIND-4.1 (20). About 35,113 particles for Cp-1–tubulin and 40,746 particles for Cp-52–tubulin were picked manually using cisTEM (21). After 2D classification, 18,899 particles for Cp-1–tubulin rings with 8-fold symmetry, 14,468 particles for Cp-1–tubulin rings with 9-fold symmetry, and 31,098 particles for Cp52 rings with 8-fold symmetry were used to generate *ab initio* 3D models and autorefinement using cisTEM. A final round of manual refinement was carried out for each structure with a soft mask. The resolution calculation was based on the gold-standard Fourier shell correlation at the 0.143 criterion. Cryo-EM data collection, refinement, and validation statistics are given in Table 1. Local resolution was calculated with ResMAP (22).

#### Model building and refinement

Model building into the Cp-1–tubulin map with 8-fold symmetry was initiated by fitting the crystal structure of human tubulin (PDB ID: 6S8L) into the map using UCSF Chimera (University of California San Francisco) (23) and real-space refinement in Phenix (24). The resulting structure was rebuilt in Coot (25) followed by multiple cycles of real-space refinement in Phenix. The final refined model was then used to build models into the Cp-52–tubulin map and Cp-1–tubulin map with 9-fold symmetry. The new models were further built into the maps using Coot, and the real space was refined by Phenix. Initial 3D models for Cp-1 and Cp-52 were obtained from PubChem (https://pubchem.ncbi.nlm.nih.gov/), and the geometry restraints and optimized structures were generated using the Phenix restraints editor especially ligands (REEL) and Phenix electronic ligand builder and optimization workbench (eLBOW). The resulting ligand structures were fitted into density in Coot and refined in Phenix.

#### MD

MD simulations were carried out using the Desmond simulation package (Schrödinger Release 2017-3). Protein and ligand were prepared by Protein Preparation Wizard, and the optimized potentials for liquid simulation force field (OPLS_2005) parameters were used in restraint minimization and system building (26). The system was set up for simulation using a predefined water model (TIP3P) as a solvent. The electrically neutral system for simulation was built with 0.15 M NaCl in 10 Å buffer. The NPT ensemble with 300 °K, and a pressure of 1 bar was applied in the run. The simulation was performed for 5 ns, and the trajectory sampling was done at an interval of 1.2 ps. The short-range coulombic interactions were analyzed using a cutoff value of 9.0 Å using the short-range method. The smooth particle mesh Ewald method was used for handling long-range coulombic interactions. The interactions between the protein and ligand were analyzed using the Simulation Interaction Diagram tool implemented in the Desmond MD package. The stability of MD simulation was monitored by RMSD of the ligand and protein atom positions in time.

#### Model assessment

Protein interfaces were examined with PDBePISA (27). Subunit stabilities and interaction energies were calculated with FoldX, as a tool within Yasarra (28). Molecular illustrations were prepared with UCSF Chimera (23). Ligand–protein interactions were analyzed by Discovery Studio (Biovia).

## Data availability

Maps and models have been deposited in the Electron Microscopy Data Bank, https://www.ebi.ac.uk/pdbe/emdb/ (accession nos. EMD-23615, EMD-23569, and EMD-23627). Models have been deposited in the PDB, https://www.ebi.ac.uk/pdbe/ (PDB ID codes: 7M18, Cp-1–tubulin C8 rings; 7LXB, Cp-52–tubulin C8 rings; 7M20, Cp-1–tubulin C9 rings).

## Supporting information

This article contains supporting information.

## Conflict of interest

The authors declare that they have no conflicts of interest with the contents of this article.
