# Supplementary material for: Conformational changes in tubulin upon binding cryptophycin-52 reveal its mechanism of action
Source: J Biol Chem. 2021 Aug 28;297(4):101138. doi: 10.1016/j.jbc.2021.101138 (PMC8456064; doi:10.1016/j.jbc.2021.101138)
Supplement: Figures S1–S6 and Table S1 [file mmc1.pdf]

Supporting Information

Conformational changes in tubulin upon binding Cryptophycin-52 reveal its mechanism of action

Elif Eren[1], Norman R. Watts[1], Dan L. Sackett[2], and Paul T. Wingfield[1]

[1]Protein Expression Laboratory, NIAMS, National Institutes of Health, Bethesda, MD 20892, USA.

[2]Division of Basic and Translational Biophysics, NICHD, National Institutes of Health, Bethesda, MD 20892, USA.

Fig. S1. Representative micrographs and 2D class averages **2**

Fig. S2. FSC curves and angular orientation distribution plots **3**

Fig. S3. Local resolution maps **4**

Fig. S4. Cp-52 and Cp-1 binding sites on tubulin **5**

Fig. S5. Cp-52 and Cp-1 binding and accessibility **6**

Fig. S6. Cp-1-induced tubulin rings composed of different numbers of heterodimers **7**

Table S1. Tubulin interface properties **8**

Movie S1. Molecular dynamics simulation of the Cp-52-tubulin binding site **8**

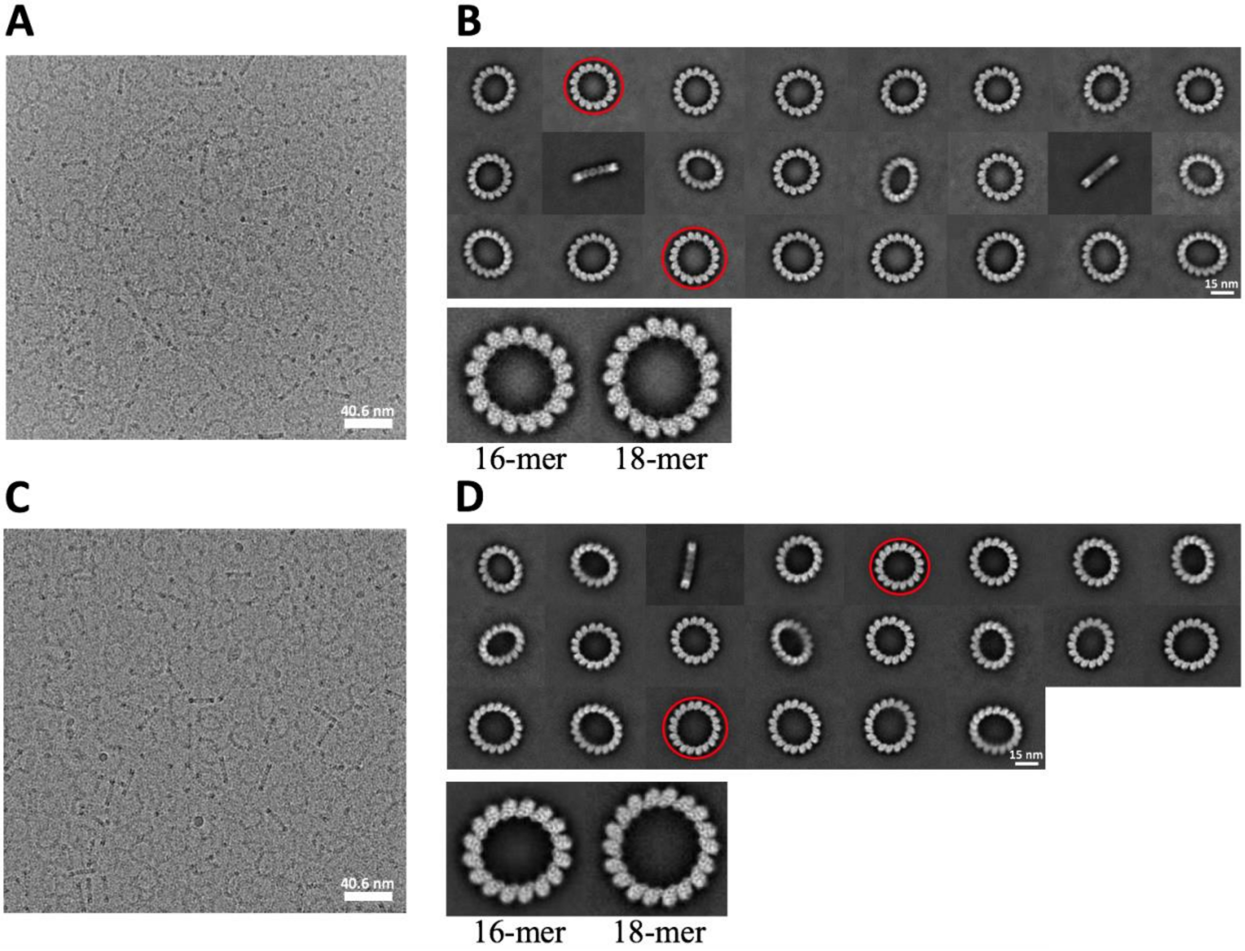

Figure S1. Representative micrographs and 2D class averages of Cp-1-tubulin and Cp-52-tubulin.
(A) Representative micrograph of Cp-1-tubulin rings.
(B) 2D class averages of Cp-1-tubulin rings. Close-up views of rings with two different sizes are shown at the bottom.
(C) Representative micrograph of Cp-52-tubulin rings.
(D) 2D class averages of Cp52-tubulin rings. Close-up views of rings with two different sizes are shown at the bottom.

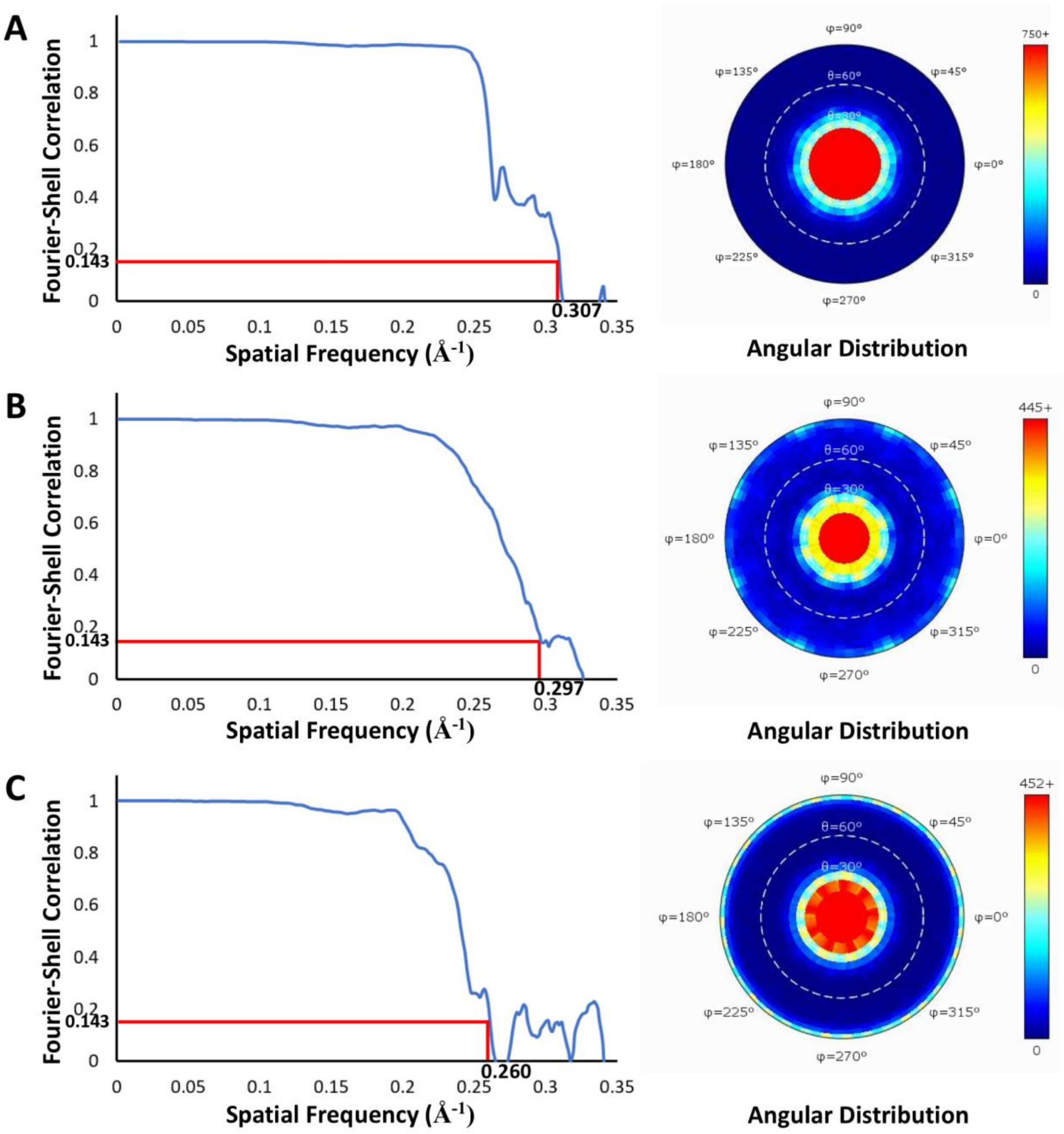

Figure S2. Gold-standard Fourier-Shell correlation (FSC) curves of final 3D reconstructions and angular orientation distribution of the particles used in the final reconstruction.
(A) Cp-52-tubulin with C8 symmetry. The resolution is ~ 3.3 Å at the FSC cutoff of 0.143.
(B) Cp-1-tubulin with C8 symmetry. The resolution is ~ 3.4 Å at the FSC cutoff of 0.143.
(C) Cp-1-tubulin with C9 symmetry. The resolution is ~ 3.9 Å at the FSC cutoff of 0.143. The particle distribution is indicated by different color shades.

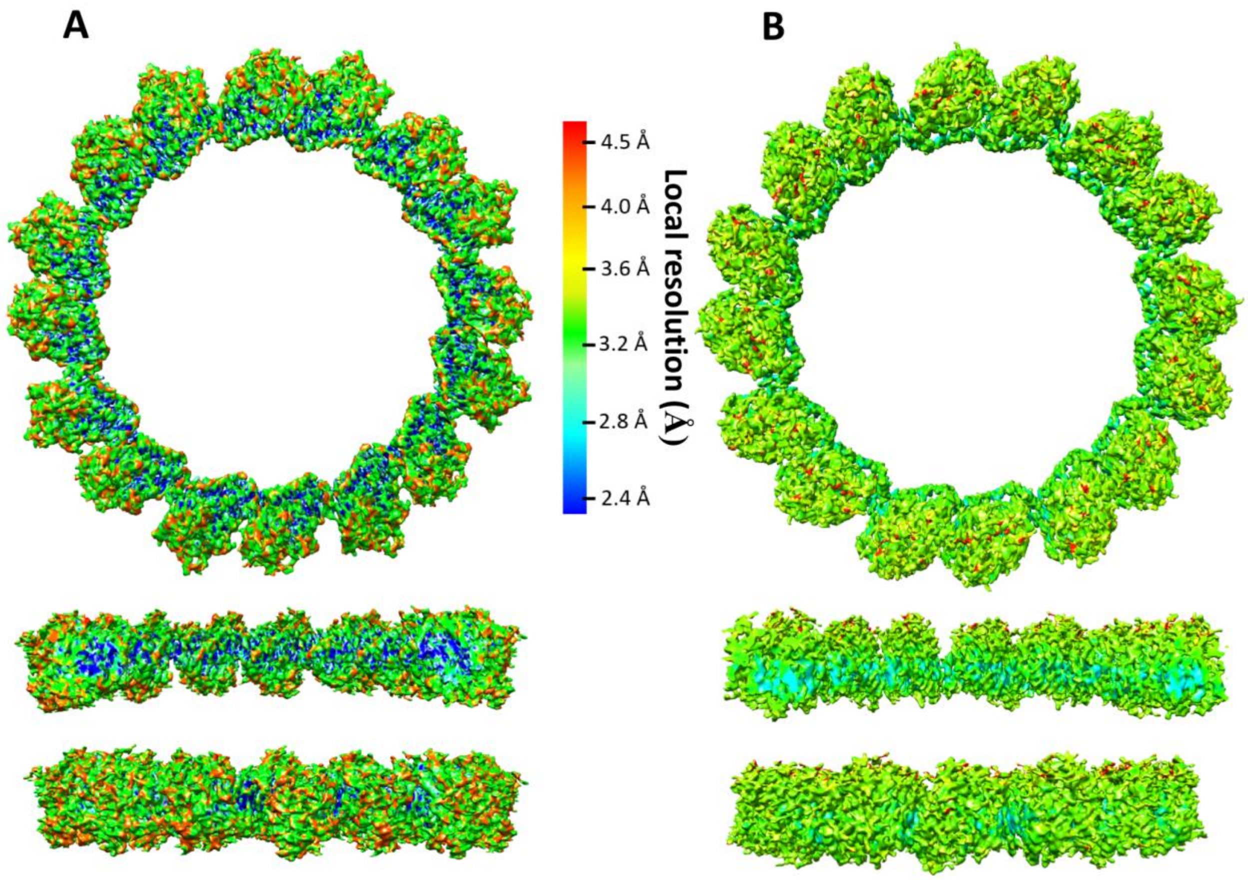

Figure S3. Local resolution maps.
(A) Cp-52-tubulin with C8 symmetry as viewed from front (top) and inside (middle) and side (bottom).
(B) Cp-1-tubulin with C8 symmetry as viewed from front (top) and inside (middle) and side (bottom).
Local resolution maps were calculated by ResMap. Color depicts local resolution as a proxy for local structure properties. Blue indicated higher resolution areas and red indicates lower resolution areas.

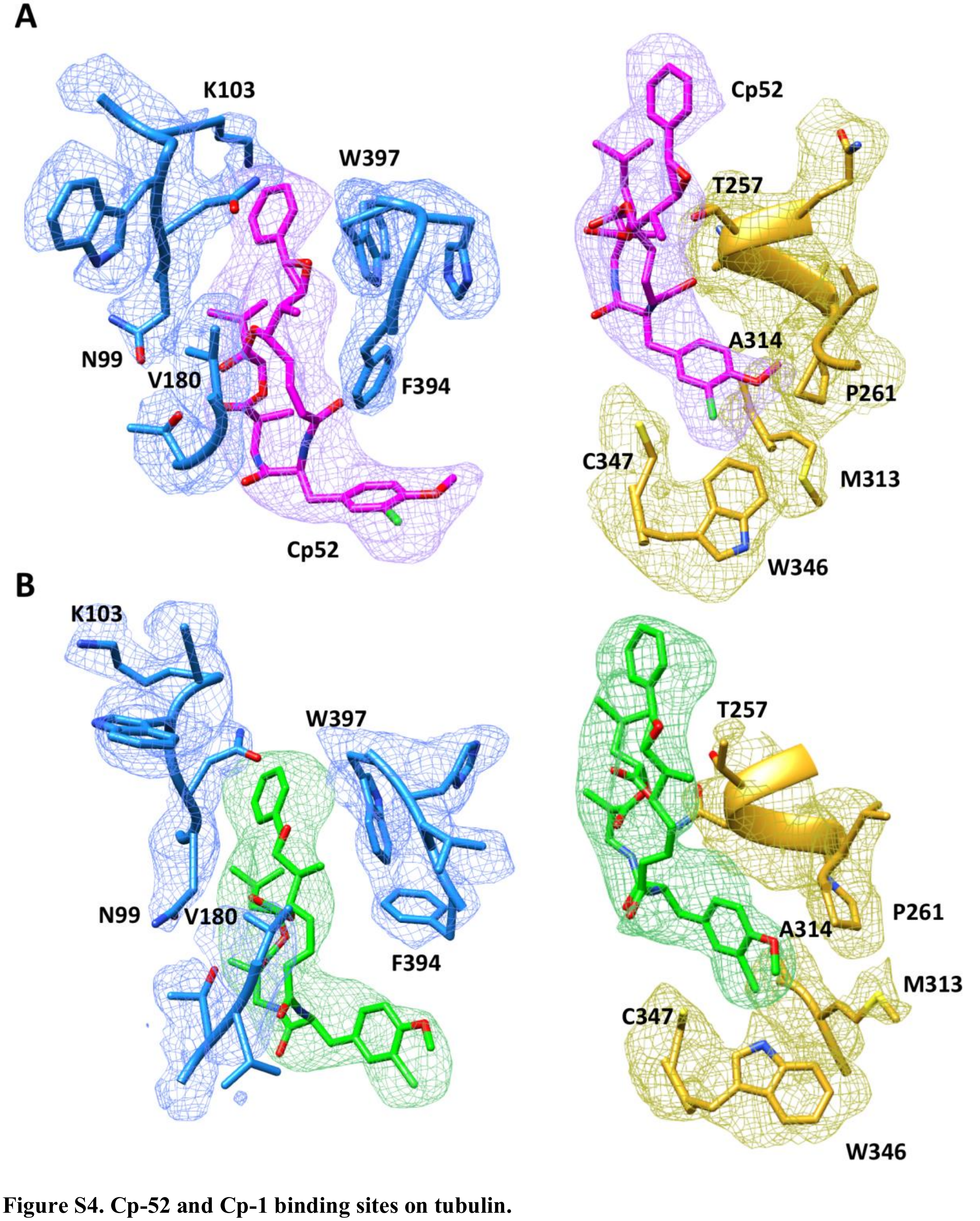

Figure S4. Cp-52 and Cp-1 binding sites on tubulin.
(A) Cp-52 binding site on β-tubulin (left) and α-tubulin (right).
(B) Cp-1 binding site on β-tubulin (left) and α-tubulin (right). Cp-52 is shown in magenta and Cp-1 is shown in green. Coordinating residues in β-tubulin and α-tubulin are shown in blue and gold, respectively. Cryo-EM densities around the drug molecules and residues located in the binding pockets are shown as a mesh representation. Residues coordinating the drug molecules are labeled.

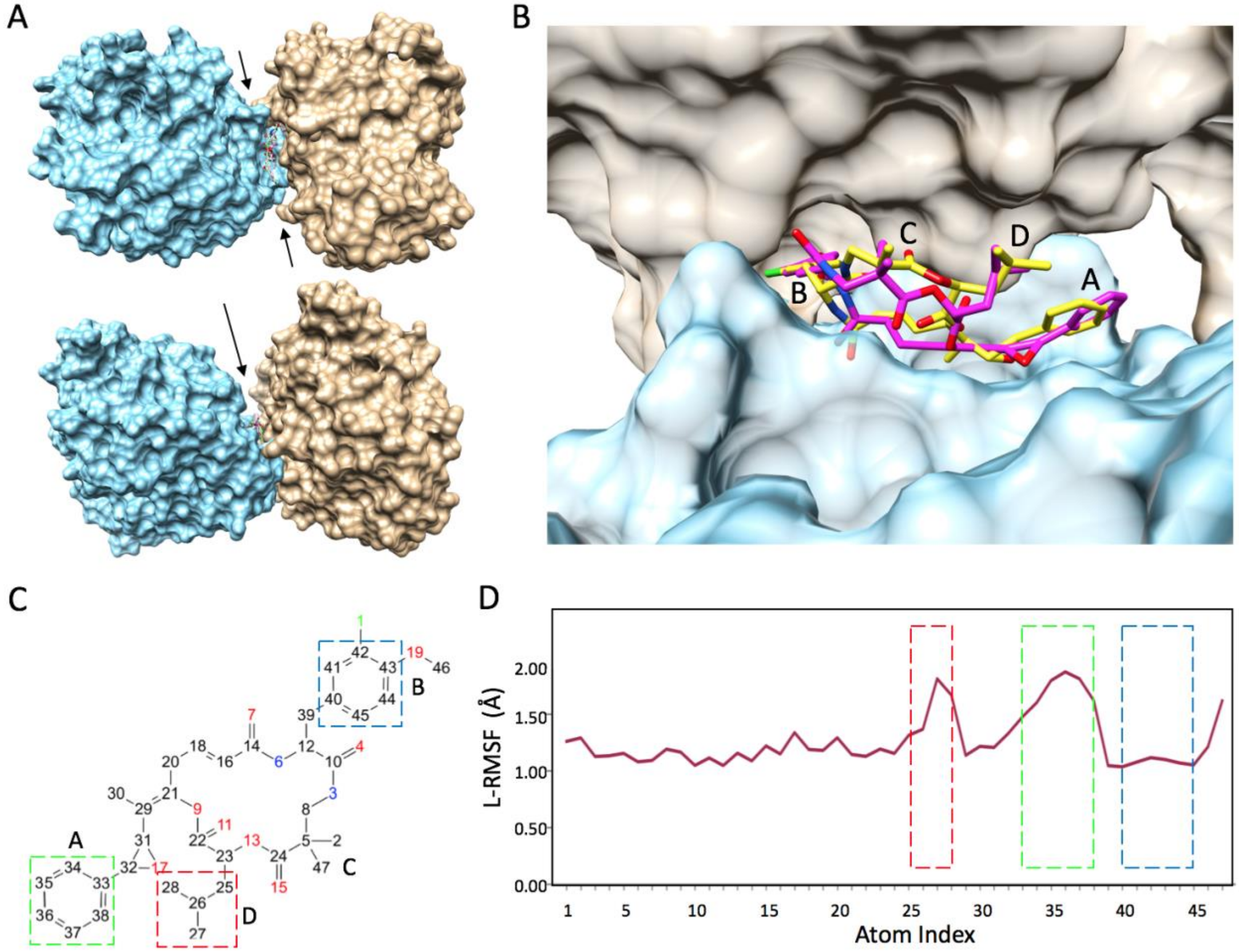

Figure S5. Cp-52 and Cp-1 binding and accessibility.
(A) Outside (upper) and lateral (lower) views of tubulin heterodimer (β-tubulin, surface, blue; α-tubulin, surface, brown) in rings, with the binding site indicated (arrows).
(B) Enlarged view of Cp-1 (stick, yellow and heteroatom) and Cp-52 (stick, magenta and heteroatom) superimposed at the inter-dimer site between β-tubulin (surface, blue) and α-tubulin (surface, brown). Units A, B, C, and D are indicated. Unit A in both Cp-1 and Cp-52 is exposed towards the outside of rings (lumen in the case of microtubules) whereas Unit B is buried in a deep pocket between the tubulin subunits. Unit C has a *gem*-dimethyl group in Cp-52 whereas it has a single methyl group in Cp-1. The Unit D isobutyl group is exposed and not involved in binding interactions in both Cp-1 and Cp-52.
(C) Structure of Cp-52.
(D) Per-atom Ligand-RMSF (L-RMSF) of Cp-52 bound to tubulin. Corresponding regions in the structure (C) and the L-RMSF profile (D) are boxed in red, green, and blue. The torsion profiles for the Unit D isobutyl 23-25 and 25-26 bond axes are both also exceptionally broad (not shown).

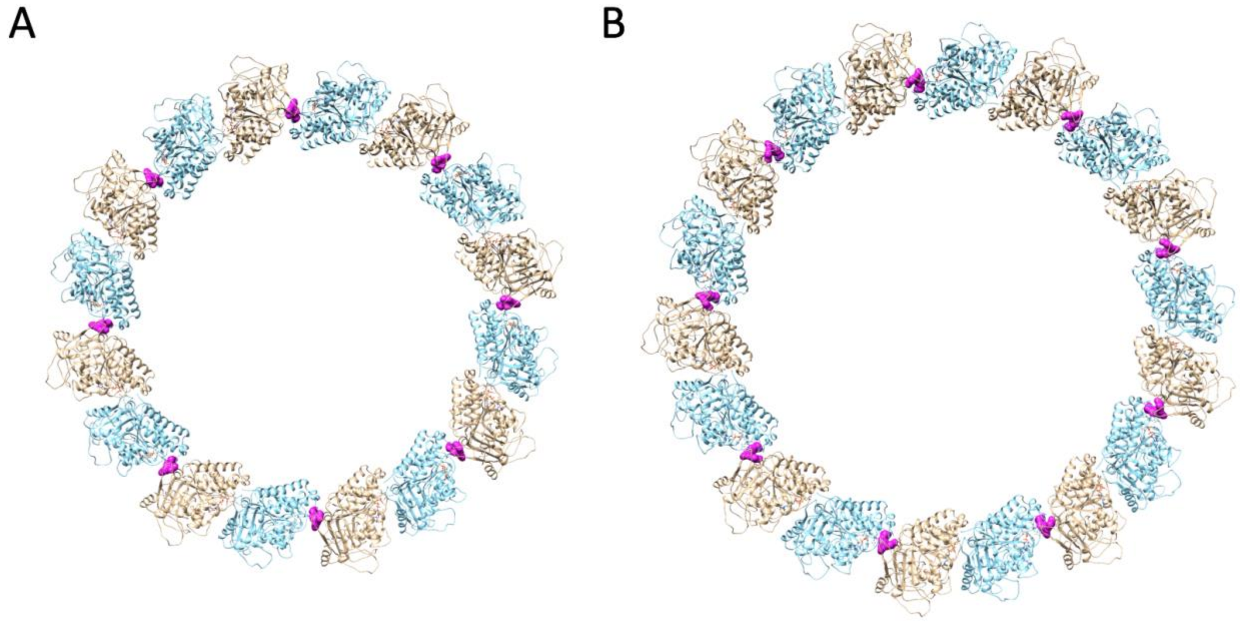

Figure S6. Cp-1-induced tubulin rings composed of different numbers of heterodimers.
(A) Ring composed of eight tubulin dimers (PDB ID: 7M18).
(B) Ring composed of nine tubulin dimers (PDB ID: 7M20). Cp-1 (surface, magenta), α-tubulin (ribbon, brown), and β-tubulin (ribbon, blue). Rings composed of seven dimers were also observed as a small minority species (not shown). The corresponding three classes of rings were also observed with Cp-52.

Table S1. Tubulin interface properties[1]

	Angle (°)[2]		Interface ($Å^2$)[3]		No. residues[3]		No. HB[3]		No. SB[3]		ΔG (kcal/mol)[6]	
	N-site	E-site	N-site	E-site	N-site[4]	E-site[5]	N-site	E-site	N-site	E-site	N-site	E-site
**Cp-1**	12.63	35.29	1171.6	519.1	30, 34	15, 18	3	2	0	0	-8.76	-3.87
**Cp-52**	12.07	35.56	1149.9	525.4	28, 37	11, 16	4	3	6	0	-15.32	-7.81
**MT[7]**	-	-	1842.4	1618.1	46, 52	48, 45	17	15	11	2	-26.87	-20.06

[1] N-site and E-site refer to the nucleotide (intra-dimer) and exchangeable (inter-dimer) sites on tubulin, respectively, and the values are the angles, interface areas, number of residues, number of hydrogen bonds, number of salt bridges, and calculated interaction energies, at each of these two sites.
[2] Values are the mean of three measurements (Cα least squares fit) relative to microtubule structures of comparable resolution (PDB ID: 5SYF, 6DPV, and 6WVR).
[3] Interface area, number of residues, hydrogen bonds, and salt bridges as determined with PDBePISA.
[4] On α-tubulin, and β-tubulin, in this order.
[5] On β-tubulin, and α-tubulin, in this order.
[6] Tubulin subunit interaction energy calculated with FoldX. Ligands (Cp-1, Cp-52, GDP, GTP, Taxol) were included when present in the structure.
[7] Values are means for three microtubules (as above), except for the ΔG values where six microtubules were used to obtain the reference mean (PDB ID: 3JAR, 6B0C, 6B0I, 6B0L, 6DPV, and 6WVR).

Movie S1. Molecular dynamics simulation of the Cp-52-tubulin binding site. α-tubulin (brown), β-tubulin (blue), Cp-52 (magenta). Interacting residues are shown as sticks for α-tubulin (yellow) and β-tubulin (green). For simplicity solvent molecules are not shown. The movie has been prepared using Maestro (Schrödinger).
